# *Klebsiella pneumoniae* carrying multiple alleles of antigen 43-encoding gene of *Escherichia coli* associated with biofilm formation

**DOI:** 10.1007/s10096-023-04552-6

**Published:** 2023-01-25

**Authors:** Martina Tambassi, Elena Passarini, Ilaria Menozzi, Melissa Berni, Chiara Bracchi, Alessandra Dodi, Luca Bolzoni, Erika Scaltriti, Marina Morganti, Giulia Ferrarini, Laura Sordi, Mario Sarti, Simone Ambretti, Stefano Pongolini

**Affiliations:** 1 Risk Analysis and Genomic Epidemiology Unit, Istituto Zooprofilattico Sperimentale Della Lombardia e Dell’Emilia-Romagna (IZSLER), Parma, Italy; 2 Department of Chemistry, Life Sciences and Environmental Sustainability, University of Parma, Parma, Italy; 3 Clinical Microbiology Unit, AUSL Modena, Baggiovara, Italy; 4 Microbiology Unit, IRCCS Azienda Ospedaliero-Universitaria Di Bologna, Bologna, Italy

**Keywords:** *Klebsiella pneumoniae*, Antigen 43, Biofilm, ST307, Colistin resistance, Mcr, Capsule

## Abstract

A clinical strain of *Klebsiella pneumoniae* typed as sequence type 307 carrying three different alleles of the *flu* gene encoding the *Escherichia coli* virulence factor antigen 43 associated with biofilm formation was detected and characterized. The *flu* alleles are located in the chromosome inside putative integrative conjugative elements. The strain displays the phenotypes associated with Ag43, i.e. bi-phasic colony morphology and enhanced biofilm production. Furthermore, the strain produces low amount of capsule known to affect Ag43 function. Analysis of 1431 worldwide deposited genomes revealed that 3.7% *Klebsiella pneumoniae* carry one or two *flu* alleles.

**Supplementary Information:**

The online version contains supplementary material available at 10.1007/s10096-023-04552-6.

## Introduction

*Klebsiella pneumoniae* represents a severe health threat worldwide because of the rapid dissemination of multidrug-resistant and hypervirulent strains due to the acquisition of drug resistance and hypervirulence genes by horizontal gene transfer. Indeed, *K. pneumoniae* has an extraordinary ability to acquire exogenous DNA, testified by its huge pangenome which includes ca. 20,000 genes [1].

An important pathogenicity trait of *K. pneumoniae* is the ability to form biofilm, a community of bacterial cells embedded in an extracellular matrix. Biofilm enables *K. pneumoniae* to colonize medical devices which become means for the entry into the human body [2]. In addition, biofilm enables bacterial tolerance to antibiotics and facilitates the transfer of genes between bacterial species [3]. The ability of *K. pneumoniae* to form biofilm is influenced by the capsule, which is differentiated in multiple capsular types.

Here, the identification and characterization of a nosocomial strain of *K. pneumoniae* (KP47) typed as sequence type 307 (ST307) and carrying three different alleles of the *flu* gene encoding the virulence determinant of *Escherichia coli* antigen 43 (Ag43) are reported [4, 5]. Ag43 is a type V autotransporter that functions as an adhesin capable of self-recognition and thus promotes self-aggregation and biofilm formation [6] potentially contributing to virulence in *K. pneumoniae*. To our knowledge, this is the first report of the *flu* gene in this species. KP47 strain was characterized for phenotypes typically associated with Ag43, i.e. double colony morphology, and enhanced biofilm production and the expression of the *flu* alleles was evaluated. The genetic location of the *flu* alleles in KP47 from its complete genome was described and the diffusion of *flu* among 1431 deposited genomes of *K. pneumoniae* was estimated.

## Materials and methods

### Bacterial strains

The strains KP47 and KP16 were isolated in the Italian hospitals Policlinico Sant’Orsola and Baggiovara, respectively, during a survey on mobile colistin-resistant genes in *Enterobacterales* [7].

### Illumina and nanopore sequencing

For Illumina sequencing, DNA was extracted using NucleoSpin® tissue kit (Macherey–Nagel), and sequencing libraries were prepared with DNA Prep, (M) tagmentation kit and sequenced on a MiSeq (Illumina Inc., San Diego, CA, USA) in a 2 × 300 bp paired-end run. For Nanopore sequencing, KP47 DNA was extracted using QIAGEN Genomic-tip 500/G kit, and libraries were prepared using ligation sequencing kit (Oxford Nanopore Technologies) and sequenced on a R9.4.1 flow cell (FLO-MIN106D) in a MinION Mk1B device.

### Genome assembly, annotation, and comparison

Illumina reads were trimmed through Trimmomatic 0.39 [8]. Nanopore reads were filtered by quality using Filtlong 0.2.1 (https://github.com/rrwick/Filtlong). Assembly was performed through Unicycler 5.0 [9]. Genomes were annotated by PROKKA 1.14.6 [10], and chromosomes were aligned by MAUVE 2.4.0 [11].

### Density-based relative quantification of capsule production

Capsule production was measured using a density-based method [12].

### Biofilm formation assay

The O’Toole protocol was used for biofilm formation assay [13].

### RNA extraction

RNA was extracted from stationary and exponential growth phases, planktonic bacterial cells recovered from wells were biofilm occurred (biofilm-planktonic phase) and biofilm-forming cells (biofilm-sessile phase). Details are provided in supplementary material.

### Gene expression analysis

Gene expression of the three *flu* alleles was evaluated by Quantitative reverse-transcription PCR (qRT-PCR). The *rpoD* gene was chosen as reference gene [14]. Primers are reported in Table S1. qRT-PCR was performed using GoTaq qPCR Master Mix (Promega) kit. Details of primer design and qRT-PCR protocol are provided in supplementary material.

### In silico detection of *flu* alleles

All complete genomes available of *K. pneumoniae* were downloaded from NCBI on May 25, 2022, and their species was verified using Kleborate 2.2.0 [15]. The sequence type (ST) of each genome was deduced from Kleborate analysis. Srst2 0.2.0 [16] was used to evaluate the similarity of *flu* alleles of KP47 to the set of *flu* alleles downloaded on April 21, 2022, from *E. coli* BIGSdb [17]. An ABRicate custom database [18] was built adding the three KP47 alleles to those present in the *E. coli* BIGSdb. The database was used to find any *flu* allele in the downloaded *K. pneumoniae* complete genomes.

### Phylogenetic analysis

A reference-based SNP analysis was carried out with SNIPPY 4.6.0 [19] on a selection of the downloaded *K. pneumoniae* genomes. The selection included one representative genome from each ST carrying at least one *flu* gene and one representative genome from the other STs provided that they included at least 5 genomes. A maximum-likelihood tree was inferred from the core-SNP matrix with RAxML 8.2.12 [20] using the GTR model and 100 bootstrap iterations.

## Results and discussion

**Fig. 1:**
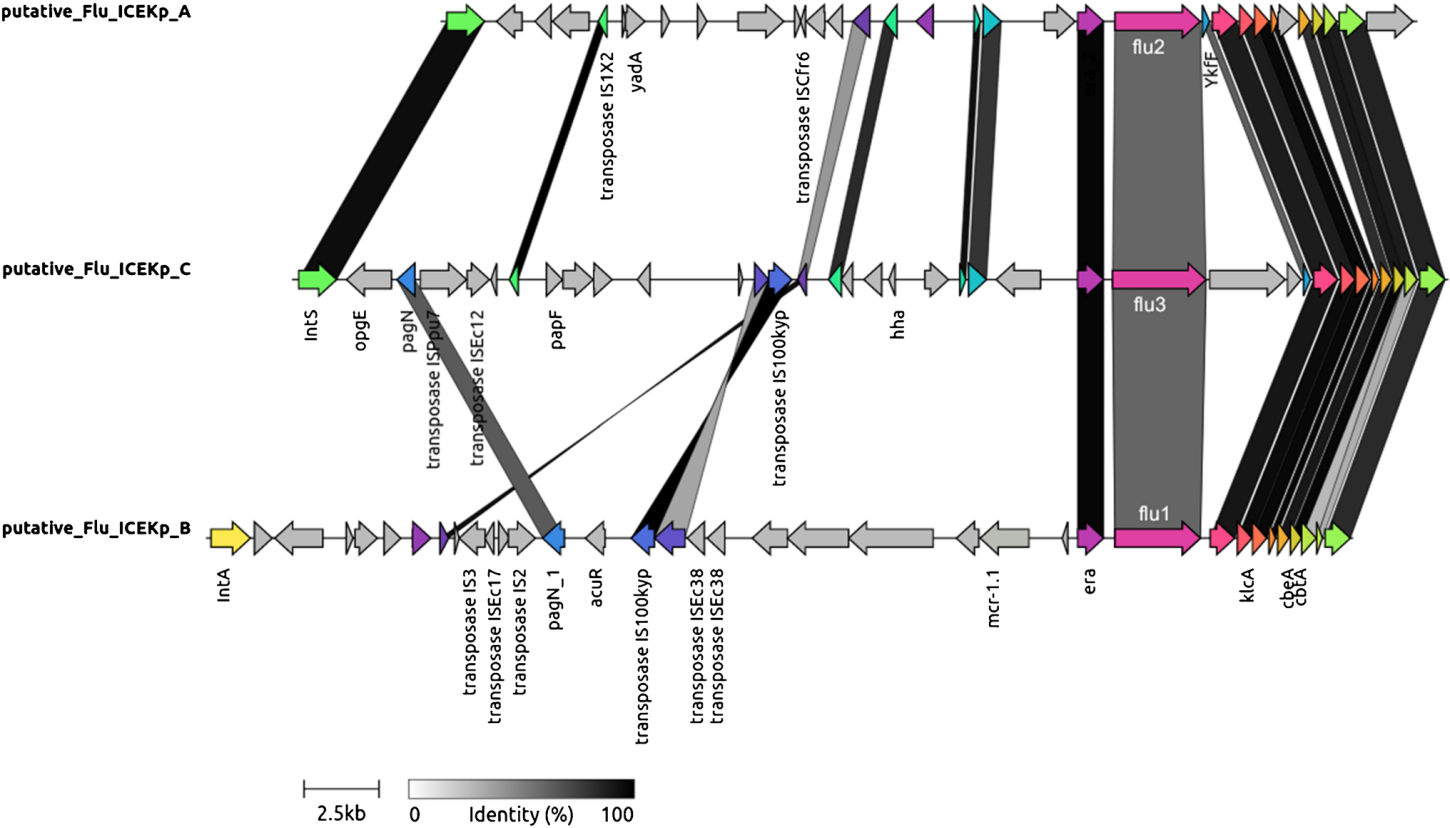
Comparison of the gene content of the three putative ICE of strain KP47. Insertion sequences, containing *flu* genes, were extracted aligning the KP47 chromosome with that of control strain KP16 and then compared to each other by using Clinker [34]. CDSs are depicted as arrows: colored CDSs are shared between at least two ICEs and genes with known function are labeled. Homologs are linked by sheets colored in grey scale depending on their nucleotide percentage of identity, as reported in the legend

KP47 strain was typed as ST307 and K-serotype KL102. ST307 is one of the epidemiologically successful clones of *K. pneumoniae*, associated with multidrug resistance and nosocomial outbreaks worldwide [21]. KP47 genome revealed three *flu* alleles in the bacterial chromosome encoding Ag43 named *flu1*, *flu2* and *flu3*, and 2847, 2847 and 3120 bp long, respectively. The average nucleotide identity of the three alleles was 79.9%. Alignment of KP47 chromosome to KP16, a *flu*-negative control strain belonging to ST307, detected the presence of three large insertions (~ 33–39 Kbp) containing the *flu* genes and located adjacent to phenylalanine tRNA or methionine tRNA depending on the presence of *IntS* or *IntA* integrase gene, respectively. No match was found with the three insertions either querying the ICEberg database [22] or running PHASTER for phage search [23]. However, putative *attL* and *attR* sites were found in the flaking regions of the insertions, suggesting they could be integrative conjugative elements (ICE) [24, 25]. The comparison of CDS regions of the putative ICEs is depicted in Fig. 1. Notably, *flu1* was found close to mobilized colistin resistance gene *mcr-1.1.* The circulation of *mcr* genes is highly monitored worldwide as colistin is a last resort antibiotic for the treatment of multidrug-resistant *Enterobacteriaceae*. The possible co-transfer of *flu1* and *mcr-1* genes through ICE represents a serious threat for public health, especially considering the epidemiological importance of ST307.

**Fig. 2:**
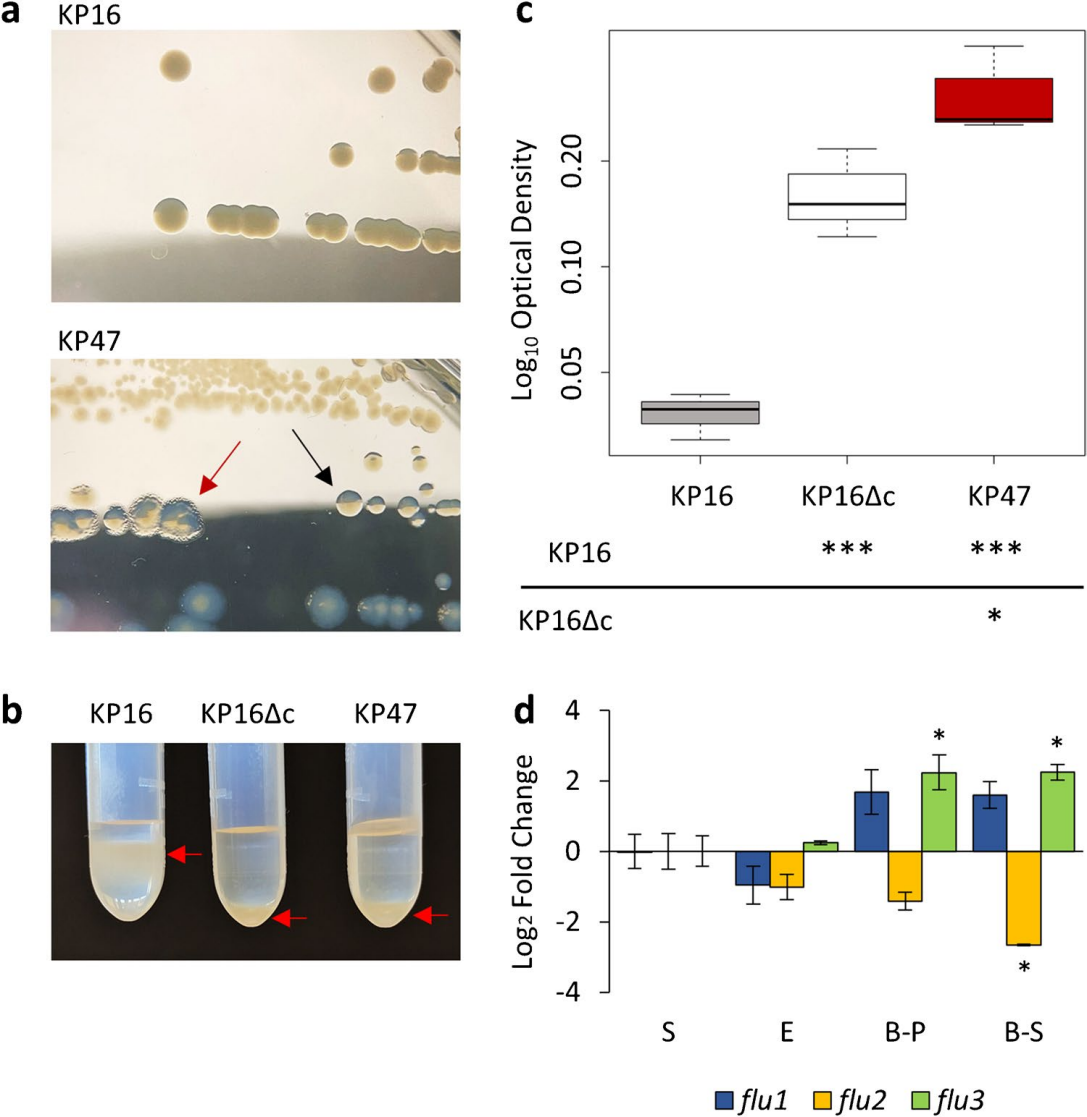
Phenotypic analysis. **a** Colony morphology. Strains were streaked on LB agar plates. KP16 carries no *flu* gene and is a high capsule producer, its colonies appear smooth, tall, circular, and opaque. KP47 carries three *flu* alleles and is a low capsule producer; it showed two colony morphologies attributable to (i) Ag43 phase OFF (black arrow), colonies are smooth, tall and circular, and translucent, and (ii) Ag43 phase ON (red arrow), colonies are large, flat, frizzy, irregular, and translucent. **b** Relative quantification of capsule production. The amount of capsule produced by the studied strains was quantified by a density-based method using 40% Percoll solution. Arrows indicates the position of bacterial cultures above (high capsule producers) or under (low capsule producers) the Percoll solution. **c** Biofilm formation. The y-axis represents the log-transformed OD_600_ values obtained by measuring the optical density of crystal-violet stained biofilms. Three biological replicates were realized, and ANOVA test was performed on the log-transformed data to fulfill the homoscedasticity requirement. The table reports *p* values from ANOVA test (*p < 0.05, ***p < 0.001). **d** Gene expression analysis. Relative expression of *flu1*, *flu2*, and *flu3* in three different growth conditions, exponential phase (E), biofilm-planktonic phase (B-P), and biofilm-sessile phase (B-S) compared to the stationary growth phase (S). Each value is the mean of three biological replicates. The error bars represent the standard error of the mean. Asterisks indicate *p* value ≤ 0.05

Expression of the *flu* gene in *E. coli* is phase-variable, leading to single strains with a heterogeneous population of colonies expressing (phase ON) and not expressing (phase OFF) Ag43 [26, 27]. Phase ON colonies are large, flat, frizzy, and irregular; phase OFF colonies are smooth, tall, and circular. The control strain KP16 showed phase OFF colonies, whereas KP47 presented both phase ON and OFF colonies (Fig. 2a) consistent with Ag43-positive *E. coli*. Furthermore, all KP47 colonies appeared translucent, whereas KP16 produced opaque colonies. Difference in opacity is related to the amount of capsule produced [28]. As capsule can mask Ag43-mediated effects on biofilm production in *K. pneumoniae* [29], capsule production of KP47 was analyzed before investigating its biofilm formation capacity. A spontaneous translucent mutant of KP16 (KP16Δc) was used as negative control for capsule production (details in supplementary) [28]. KP47, as well as KP16Δc, produced less capsule than KP16 (Fig. 2b). Genetic analysis of genes encoding the biosynthesis machinery of the capsular polysaccharides revealed non-synonymous mutations in *wcuH* (G815C, arginine-threonine) and *wzc* (C1745T, serine-phenylalanine) genes of KP47 compared to KP16 together with a long insertion inside *wbaP* gene (Fig. S1). Mutations in *wzc* as well as *wbaP* are known to impair capsule production [28, 30].

KP47 was then tested for the ability to form biofilm compared to KP16. KP16Δc was included in the analysis to evaluate biofilm formation also in comparison with a low capsule producer, because capsule can impair biofilm formation [31]. As expected, the amount of biofilm produced by KP16Δc was significantly higher than KP16 (Fig. 2c). KP47 resulted the strongest biofilm producer. Identification of strains with reduced capsule production associated with an aggregation factor like Ag43 and increased biofilm formation could be indicative of the evolution of these strains to cause localized persistent infections [30].

Transcripts of the three *flu* alleles were detected in all conditions studied, indicating that these genes are actually expressed by KP47. The expression profiles of *flu1* and *flu3* were similar. However, the expression of *flu1* was not statistically different in the tested conditions compared to the stationary phase, while *flu3* was significantly upregulated both in biofilm-planktonic and sessile phases. Differently, *flu2* was significantly downregulated during biofilm formation (Fig. 2d).

**Fig. 3:**
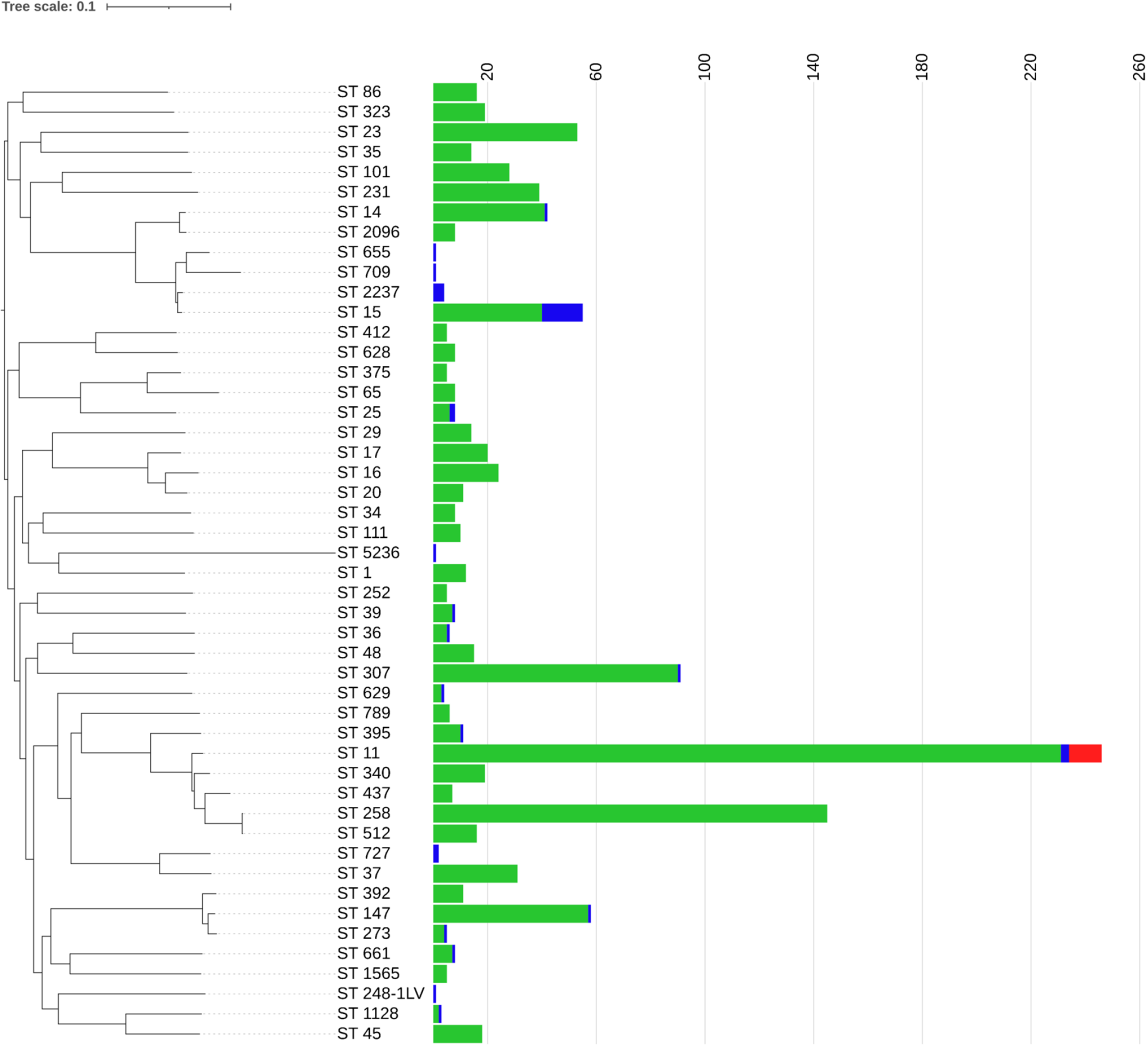
Spread of *flu* genes among different STs. A reference-based SNP analysis was carried out on assemblies of selected *K. pneumoniae* genomes out of 1431 closed genomes available. The selection included one representative genome from each ST carrying at least one *flu* gene, and one representative genome from the other STs provided that they included at least 5 genomes. Strain NTUH-K2044 (NC_012731.1) of ST23 was used as reference genome [35]. The ML tree based on core SNPs shows phylogenetic relationships between STs. Bars indicate the number of genomes without *flu* genes (green), carrying one *flu* allele (blue) and two *flu* alleles (red)

The *flu* alleles of KP47 were compared to those of the database downloaded from *E. coli* BIGSdb. Percent identity was 99.51% for *flu1* with allele 2570, 99.75% for *flu2* with allele 2661, and 99.81% for *flu3* with allele 293. Out of the 1431 available complete genomes of *K. pneumoniae*, 53 carried at least one *flu* allele (3.7%), and 13 out of the 53 contained two *flu* alleles (0.9%) (Table S2). All *flu* alleles detected were located on the bacterial chromosome with one exception of plasmid location. The phylogenetic analysis (Fig. 3) showed that genomes carrying at least one *flu* gene belonged to different STs in very distant branches and that not all the genomes belonging to a specific ST carried *flu* genes. This evidence suggests that *K. pneumoniae* likely acquired *flu* genes through several independent events of horizontal gene transfer.

This is the first report of *K. pneumoniae* carrying the *flu* gene of *E. coli*. Ag43 is involved in many steps of *E. coli* pathogenesis, such as biofilm formation but also uptake and survival in polymorphonuclear neutrophils [32] and persistence in urinary tract [33]. Therefore, the acquisition of *flu* by *K. pneumoniae* represents a serious threat to human health that needs to be further investigated.

## Supplementary Information

Below is the link to the electronic supplementary material.

Supplementary file1 (PDF 698 KB)

## Data Availability

New sequence data generated in this study are available from NCBI under BioProject accession no. PRJEB55839. The 1447 K*. pneumoniae* complete genomes available on May 25, 2022, were downloaded from NCBI. *E. coli flu* alleles were downloaded on April 21, 2022, from *E. coli* BIGSdb. The authors confirm all supporting data, code, and protocols have been provided within the article or through supplementary data files (two supplementary figures and four supplementary tables).
