# Supplementary material for: Klebsiella pneumoniae carrying multiple alleles of antigen 43-encoding gene of Escherichia coli associated with biofilm formation
Source: Eur J Clin Microbiol Infect Dis. 2023 Jan 25;42(3):371–7. doi: 10.1007/s10096-023-04552-6 (PMC9899198; doi:10.1007/s10096-023-04552-6)
Supplement: Supplementary file 1 — Supplementary file1 (PDF 698 KB) [file 10096_2023_4552_MOESM1_ESM.pdf]

Klebsiella pneumoniae carrying multiple alleles of Antigen 43-encoding gene of *Escherichia coli* associated with biofilm formation

Martina Tambassi,[a]# Elena Passarini,[a,b] Ilaria Menozzi,[a] Melissa Berni,[a] Chiara Bracchi,[a] Alessandra Dodi,[a] Luca Bolzoni,[a] Erika Scaltriti,[a] Marina Morganti,[a] Giulia Ferrarini,[c] Laura Sordi,[c] Mario Sarti,[c] Simone Ambretti,[d] Stefano Pongolini[a]

[a] Risk Analysis and Genomic Epidemiology Unit, Istituto Zooprofilattico Sperimentale della Lombardia e dell'Emilia-Romagna (IZSLER), Parma, Italy

[b] Department of Chemistry, Life Sciences and Environmental Sustainability, University of Parma, Parma, Italy

[c] Clinical Microbiology Unit, AUSL Modena, Baggiovara, Italy

[d] Microbiology Unit, IRCCS Azienda Ospedaliero-Universitaria di Bologna, Bologna, Italy

Supplementary material

This file contains:

Fig. S1

Table S1

Table S2

Supplementary methods

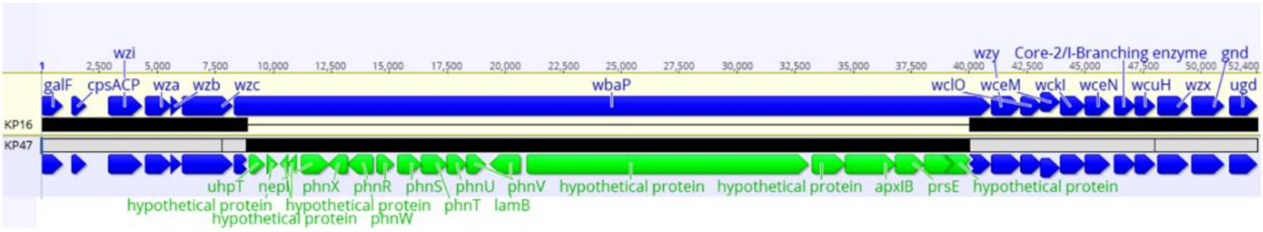

Fig. S1. CPS cluster of KP47 and KP16 strains. Sequence of CPS cluster of KP47 was aligned to that of KP16 control strain. CPS genes are displayed in blue, genes belonging to the insertion in *wbaP* gene are displayed in green.

Table S1. Primers used for qRT-PCR reactions.

GENE	SEQUENCE (5'-3')
flu1	FW: CTGAATGGCGATGTGGTCAG<br>RV: CCCTGACCGGTGAAGTTAC
flu2	FW: GTGATACCGGGCAGTTTGTTC<br>RV: GCCGGCATAAACCACAGTG
flu3	FW: CATTTCACCTCCTCACGCAC<br>RV: ATTGTTCTGCGCCATATCCG
wza	FW: ACGTGGTTGAACTCCCAGAT<br>RV: AGTACGTCACCAACCCCAAT
wzy	FW: GGAACAATGTGGACCGGTTT<br>RV: ACCCAATCACCATCATCATCA
rpoD	FW: CAACCGTATCTCCCGTCAGA<br>RV: GGCTCTTTGGCGATCTTCAG

Table S2. List of complete genomes carrying at least one *flu* gene and related information about the position on the genome, size and allele number (with % of coverage and identity) for each *flu* gene detected.

Genome ID	MLST	Start	End	Flu size (bp)	% Coverage	% Identity	Flu Allele
GCA_009914255_1_ASM991425v1	11	708842	711688	2847	100.00	99.97	Ecoli_Flu_b2000_2727
GCA_013378155_1_ASM1337815v1		698128	700974	2847	100.00	99.97	Ecoli_Flu_b2000_2727
		4804040	4806886	2847	100.00	99.26	Ecoli_Flu_b2000_2713
GCA_013378175_1_ASM1337817v1		698128	700974	2847	100.00	99.97	Ecoli_Flu_b2000_2727
		4804287	4807133	2847	100.00	99.26	Ecoli_Flu_b2000_2713
GCA_013378195_1_ASM1337819v1		698128	700974	2847	100.00	99.97	Ecoli_Flu_b2000_2727
		4804470	4807316	2847	100.00	99.26	Ecoli_Flu_b2000_2713
GCA_013378215_1_ASM1337821v1		698128	700974	2847	100.00	99.97	Ecoli_Flu_b2000_2727
		4805247	4808093	2847	100.00	99.26	Ecoli_Flu_b2000_2713
GCA_013378235_1_ASM1337823v1		698128	700974	2847	100.00	99.97	Ecoli_Flu_b2000_2727
		4804470	4807316	2847	100.00	99.26	Ecoli_Flu_b2000_2713
GCA_013378295_1_ASM1337829v1		698128	700974	2847	100.00	99.97	Ecoli_Flu_b2000_2727
		4804041	4806887	2847	100.00	99.26	Ecoli_Flu_b2000_2713
GCA_013378315_1_ASM1337831v1		698128	700974	2847	100.00	99.97	Ecoli_Flu_b2000_2727
		4804107	4806953	2847	100.00	99.26	Ecoli_Flu_b2000_2713
GCA_013416085_1_ASM1341608v1		698128	700974	2847	100.00	99.97	Ecoli_Flu_b2000_2727
		4804040	4806886	2847	100.00	99.26	Ecoli_Flu_b2000_2713
GCA_013416105_1_ASM1341610v1		698128	700974	2847	100.00	99.37	Ecoli_Flu_b2000_2727
		4803306	4806152	2847	100.00	99.26	Ecoli_Flu_b2000_2713
GCA_013416125_1_ASM1341612v1		4766448	4769294	2847	100.00	99.26	Ecoli_Flu_b2000_2713
GCA_022353825_1_ASM2235382v1		4260813	4263659	2847	100.00	100.00	Ecoli_Flu_b2000_2882
GCA_022354545_1_ASM2235454v1		726106	728952	2847	100.00	99.97	Ecoli_Flu_b2000_2727
		4812406	4815252	2847	100.00	99.33	Ecoli_Flu_b2000_2713
GCA_022493975_1_ASM2249397v1		726106	728952	2847	100.00	99.97	Ecoli_Flu_b2000_2727
		4811149	4813995	2847	100.00	99.33	Ecoli_Flu_b2000_2713
GCA_902650055_1_Kp_294_4		5469292	5472138	2847	100.00	99.97	Ecoli_Flu_b2000_2727
GCA_001521895_1_ASM152189v1	14	3016076	3018922	2847	100.00	96.52	Ecoli_Flu_b2000_2740
GCA_002209405_1_ASM220940v1	15	2392646	2395492	2847	100.00	99.33	Ecoli_Flu_b2000_2713
GCA_003054385_1_ASM305438v1		1202564	1205410	2847	100.00	99.33	Ecoli_Flu_b2000_2713
GCA_004295385_1_ASM429538v1		12885	15731	2847	100.00	99.33	Ecoli_Flu_b2000_2713
GCA_004295605_1_ASM429560v1		743870	746716	2847	100.00	99.89	Ecoli_Flu_b2000_2796
GCA_004319525_1_ASM431952v1		3407076	3409922	2847	100.00	99.33	Ecoli_Flu_b2000_2713
GCA_011769825_1_ASM1176982v1		752947	755793	2847	100.00	99.33	Ecoli_Flu_b2000_2713
GCA_012970325_1_ASM1297032v1		4857008	4859854	2847	100.00	99.33	Ecoli_Flu_b2000_2713
GCA_012970685_1_ASM1297068v1		4721606	4724452	2847	100.00	99.33	Ecoli_Flu_b2000_2713
GCA_012971665_1_ASM1297166v1		4713977	4716823	2847	100.00	99.33	Ecoli_Flu_b2000_2713
GCA_014123345_1_ASM1412334v1		697105	699592	2488	87.39	99.96	Ecoli_Flu_b2000_2727
GCA_022354365_1_ASM2235436v1		2293818	2296638	2821	99.09	99.97	Ecoli_Flu_b2000_2727
GCA_022434315_1_ASM2243431v1		4671938	4674784	2847	100.00	99.33	Ecoli_Flu_b2000_2713
GCA_023066625_1_ASM2306662v1		4689982	4692828	2847	100.00	99.30	Ecoli_Flu_b2000_2713
GCA_023066645_1_ASM2306664v1		4649623	4652469	2847	100.00	99.33	Ecoli_Flu_b2000_2713
GCA_023205035_1_ASM2320503v1		786564	789410	2847	100.00	98.42	Ecoli_Flu_b2000_2599
GCA_003812105_1_ASM381210v1	25	221499	224345	2847	100.00	98.03	Ecoli_Flu_b2000_2653
GCA_014788765_1_ASM1478876v1		4920267	4923113	2847	100.00	98.03	Ecoli_Flu_b2000_2653
GCA_022964795_1_ASM2296479v1	36	4809881	4812727	2847	100.00	99.37	Ecoli_Flu_b2000_2713
GCA_022354125_1_ASM2235412v1	39	102299	105145	2847	100.00	100.00	Ecoli_Flu_b2000_2619
GCA_021166135_1_ASM2116613v1	147	4704585	4707431	2847	100.00	99.33	Ecoli_Flu_b2000_2713
GCA_018314115_1_ASM1831411v1	273	4723748	4726594	2847	100.00	99.33	Ecoli_Flu_b2000_2713
GCA_011045775_1_ASM1104577v1	395	3970356	3973202	2847	100.00	99.97	Ecoli_Flu_b2000_2727
GCA_015278095_1_ASM1527809v1	629	1817514	1820360	2847	100.00	99.02	Ecoli_Flu_b2000_2713
GCA_014169335_1_ASM1416933v1	655	4657655	4660501	2847	100.00	99.33	Ecoli_Flu_b2000_2713
GCA_022354665_1_ASM2235466v1	661	4557997	4560843	2847	100.00	99.33	Ecoli_Flu_b2000_2713
GCA_022353525_1_ASM2235352v1	709	4166010	4168856	2847	100.00	99.93	Ecoli_Flu_b2000_2814
GCA_020023465_1_ASM2002346v1	727	3510248	3513094	2847	100.00	99.33	Ecoli_Flu_b2000_2713
		4866144	4868990	2847	100.00	98.17	Ecoli_Flu_b2000_2639
GCA_020172745_1_ASM2017274v1		4771716	4774562	2847	100.00	99.33	Ecoli_Flu_b2000_2713
		707701	710547	2847	100.00	98.17	Ecoli_Flu_b2000_2639
GCA_017310405_1_ASM1731040v1	1128	735326	738172	2847	100.00	100.00	Ecoli_Flu_b2000_2844
GCA_018454365_1_ASM1845436v1	2237	856669	859156	2488	87.39	99.96	Ecoli_Flu_b2000_2727
GCA_019823785_1_ASM1982378v1		929790	932277	2488	87.39	99.96	Ecoli_Flu_b2000_2727
GCA_019823895_1_ASM1982389v1		862183	864670	2488	87.39	99.96	Ecoli_Flu_b2000_2727
GCA_020080125_1_ASM2008012v1		934516	937003	2488	87.39	99.96	Ecoli_Flu_b2000_2727
GCA_019038575_1_ASM1903857v1	5236	4812245	4815091	2847	100.00	98.31	Ecoli_Flu_b2000_2599
GCA_022494335_1_ASM2249433v1	NA	4118085	4120932	2848	100.00	99.19	Ecoli_Flu_b2000_2727
GCA_022809875_1_ASM2280987v1	ST248-1LV	137916	140765	2850	100.00	98.21	Ecoli_Flu_b2000_2664
GCA_017639065_1_ASM1763906v1	307	723900	726746	2847	100.00	98.42	Ecoli_Flu_b2000_2599
KP47		680554	683400	2847	100.00	99.51	Ecoli_Flu_b2000_2570
		765420	768266	2847	100.00	97.75	Ecoli_Flu_b2000_2661
		4999178	5002297	3120	100.00	99.81	Ecoli_Flu_b2000_293
KP34		1584741	1587587	2847	100.00	97.75	Ecoli_Flu_b2000_2661
		1669606	1672452	2847	100.00	99.51	Ecoli_Flu_b2000_2570
		2850808	2853927	3120	100.00	99.81	Ecoli_Flu_b2000_293

Supplementary methods

RNA extraction. For the stationary phase condition, strains were incubated in 3 ml of LB overnight at 37°C with shaking and RNA extraction was performed on 100 µl of bacterial culture. For the exponential phase condition, overnight cultures were diluted at OD_{600} 0.1 and, after 100 min of incubation at 37°C with shaking, 1 ml of culture was used for RNA extraction. For biofilm-planktonic and biofilm-sessile phases, strains were inoculated in 3 ml of LB broth and incubated overnight at 37°C with shaking. The following day cultures were diluted as for the biofilm formation assay and incubated for 24h at 37°C in a 96-well polystyrene plate with round bottom. Concerning planktonic cells, 100 µl of bacterial culture per strain were collected for RNA extraction, whereas biofilm cells were washed twice with 200 µl of PBS and resuspended in 100 µl of PBS by scraping the bottom of the well with pipette tips. For each strain, biofilm-forming cells were collected from eight wells to obtain enough cells for RNA extraction. Bacterial cultures were transferred in 1.5 ml tube and 2 volumes of RNAprotect Bacteria Reagent were added (QIAGEN). After vortexing and incubating at room temperature for 5 min, samples were centrifuged for 10 min at 5000 x g, the supernatant discarded and enzymatic lysis of the pellet was performed for 10 min at 37°C with 1 mg/ml of lysozyme (Sigma-Aldrich) in Tris-EDTA. The extraction was carried out using Nucleospin Macherey Nagel RNA extraction kit following manufacture's protocol. Both in-column and in-solution DNase digestion was performed. RNA was precipitated by mixing with sodium acetate (0.1 Volume, 1M, Thermo-scientific), ethanol (2.5 Volumes, 100%, Sigma-Aldrich) and glycogen (1 µl, 20 mg/ml, Invitrogen). After overnight incubation at -20°C, RNA was washed twice with 750 µl of 100% ice-cold ethanol and resuspended in water. RNA concentration and purity (A_{260}/A_{280}) were determined with a Synergy H1 Hybrid spectrophotometer (BioTek) equipped with Gen5 software. RNA extraction was done from three independent biological replicates. The extracted RNA was reverse transcribed using High-Capacity cDNA Reverse Transcription Kit (Applied Biosystems).

qPCR Primer design and details. Primers for *flu* alleles were designed to be allele specific. For this purpose, the three alleles were aligned by ClustalW 2.1 [1] and point mutations specific of each allele

were found. Allele specific-primers were designed such that the last and the second or the third to last base at primer 3′ end were positioned at SNPs sites and checked for quality through Primer3 version 4.1.0 [2]. To determine qPCR reaction efficiency, a standard curve for each primer pair was run in triplicate. Relative quantification of gene expression was done by Pfaffl method [3] and statistical analysis was done with the Student's t test ($P \leq 0{,}05$; two-tailed, unpaired). Amplicons of *flu* alleles were Sanger sequenced to confirm PCR specificity.

Isolation of a spontaneous translucent KP16 mutant. It was previously observed that *K. pneumoniae* strains can exhibit opaque, capsulated colonies with translucent segments, corresponding to spontaneous mutants which produce less capsule than the wildtype strain due to accumulated insertion sequences, point mutations or small insertion–deletion mutations on CPS genes. The ability of capsulated colonies to produce translucent segments is dependent on the incubation time (> 24 h) [4]. Therefore, to isolate a spontaneous KP16 mutant with impaired ability to produce capsule was isolated from translucent segments of KP16 colonies generates by streaking, KP16 strain was streaked on Luria Bertani (LB) agar and incubated at 37°C until bacterial colonies with translucent segments were observed. A KP16 mutant (KP16Δc) was isolated by streaking a translucent colony segment on LB agar to obtain single colonies and re-streaking a single colony twice more. KP16Δc was then Illumina sequenced to find the mutation responsible for reduced capsule production. KP16Δc reads were assembled by Unicycler 5.0 [5] and KP16 and KP16Δc assemblies were aligned by MAUVE 2.4.0 [6]. The alignment was observed at the CPS cluster level to find point mutations, indels or other insertions in CPS genes. We found that KP16Δc carried a single-base deletion generating a frameshift mutation in *wbaP* gene.

Supplementary References

1. Larkin MA, Blackshields G, Brown NP, Chenna R, McGettigan PA, McWilliam H, Valentin F, Wallace IM, Wilm A, Lopez R, Thompson JD, Gibson TJ, Higgins DG. 2007. Clustal W and Clustal X version 2.0. Bioinformatics 23:2947-2948. DOI: 10.1093/bioinformatics/btm404

2. Koressaar T, Lepamets M, Kaplinski L, Raime K, Andreson R and Remm M. 2018. Primer3_masker: integrating masking of template sequence with primer design software. Bioinformatics 34(11):1937-1938. DOI: 10.1093/bioinformatics/bty036

3. Pfaffl MW. 2001. A new mathematical model for relative quantification in real-time RT–PCR. Nucleic Acids Res 29(9):e45. DOI: 10.1093/nar/29.9.e45

4. Chiarelli A, Cabanel N, Rosinski Chupin I, Zongo PD, Naas T, Bonnin RA, Glaser P. 2020. Diversity of mucoid to non-mucoid switch among carbapenemase-producing *Klebsiella pneumoniae*. BMC Microbiol 20:325. DOI: 10.1186/s12866-020-02007-y

5. Wick RR, Judd LM, Gorrie CL, Holt KE. 2017. Unicycler: resolving bacterial genome assemblies from short and long sequencing reads. PLoS Comput Biol 13: e1005595. DOI: 10.1371/journal.pcbi.1005595

6. Darling AC, Mau B, Blattner FR, Perna NT. 2014. Mauve: Multiple alignment of conserved genomic sequence with rearrangements. Genome Res 14:1394–1403. DOI: 10.1101/gr.2289704
